# Mortality, Recruitment and Change of Desert Tree Populations in a Hyper-Arid Environment

**DOI:** 10.1371/journal.pone.0000208

**Published:** 2007-02-14

**Authors:** Gidske L. Andersen, Knut Krzywinski

**Affiliations:** Department of Biology, University of Bergen, Bergen, Norway; University of Sheffield, United Kingdom

## Abstract

**Background:**

Long-term vegetation changes in hyper-arid areas have long been neglected. Mortality, recruitment and change in populations of the ecologically and culturally important and drought persistent *Acacia tortilis* and *Balanites aegyptiaca* are therefore estimated in the Eastern Desert of Egypt, and are related to the primary agents of change, water conditions and human intervention.

**Methodology:**

A change analysis using high-resolution Corona images (1965) in combination with field data (2003) is the basis for recruitment, mortality and change estimates. For assessing the influence of water conditions on patterns in recruitment and survival, different types of generalized linear models are tested.

**Conclusions:**

The overall trend in population size in that part of the Eastern Desert studied here is negative. At some sites this negative trend is alarming, because the reduction in mature trees is substantial (>50%) at the same time as recruitment is nearly absent. At a few sites there is a positive trend and better recruitment. Frequent observations of sprouting in saplings indicate that this is an important mechanism to increase their persistence. It is the establishment itself that seems to be the main challenge in the recruitment process. There are indications that hydrological variables and surface water in particular can explain some of the observed pattern in mortality, but our results indicate that direct human intervention, i.e., charcoal production, is the main cause of tree mortality in the Eastern Desert.

## Introduction

Although vegetation changes in African drylands, i.e. desertification, have been recurrently discussed [1]–[3], changes in hyper-arid areas have long been neglected [4]. In hyper-arid areas [5] tree populations are a main indicator of long-term vegetation changes because trees are long-lived and drought-enduring. For the same reasons trees are also the drought insurance for desert dwellers and as a resource they constitute a main pillar in the traditional nomadic lifestyle [6]. High mortality in desert trees combined with lack of recruitment has been reported from arid and hyper-arid regions in Africa and the Middle East [6]–[10]. Over time such a trend will endanger tree populations. Few studies, however, monitor tree populations over longer time intervals, and little is therefore known about their temporal trends.

Recruitment is a key process for maintaining sustainable tree populations, but throughout arid lands it seems to be an infrequent event [10]–[12]. Seedlings die off during extended periods of moisture deficits [8], [13]. Even if they succeed in establish themselves, they remain under growth suppression from browsing and droughts during the sapling stage [11], [12], [14].

Tree mortality in the area considered here, the Eastern Desert of Egypt (ED), is historically known to be influenced by charcoal production [6]. Travellers in the late 19^th^ and early 20^th^ centuries AD reported the local nomads' (the Ababda) skills as charcoal producers, and some even commented on the severe effects of that activity [15]–[17]. Charcoal production is onoing in the ED also today, but it is unknown whether this is a main cause for present mortality. In the Negev, Israel, recent tree mortality seems to be mainly associated with water stress, while in the Red Sea Hills, Sudan, felling for human use, in particular for charcoal production, is the main cause [6], [7], [18].

For the successful monitoring and assessment of trends in tree populations over longer time intervals baseline historical data is imperative. However, such baseline data are often lacking. Therefore, remote sensing archives have been widely used in studies of vegetation change. However, the individual trees scattered in the desert are impossible to detect in low resolution remotely sensed data, e.g. NOAA AVHRR and MODIS, hitherto a main tool for monitoring vegetation change across African drylands. These time series data reflect mainly ephemeral herbs' greening and disappearance within short-term rainfall fluctuations [19]–[21]. Even medium resolution LANDSAT MSS and TM imagery are in many cases too coarse to detect scattered trees in hyper-arid areas or to separate trees from ephemeral plant cover [22]. High resolution data in locally focused studies together with detailed, well-positioned ground truth data is therefore needed to reveal the high spatial variability and the complex processes normally taking place [23], [24].

The overall objective of this study is to increase the information available on vegetation changes in hyper-arid regions, particularly on changes in the ecologically and culturally important tree species *Acacia tortilis* (Forssk.) Hayne and *Balanites aegyptiaca* (L.) Del. Therefore we focus on the hyper-arid ED where neither the degree nor the extent of changes in tree population has ever been quantified. For this we apply high-resolution imagery from the US reconnaissance satellite corona (1965) in combination with field inventory data (2003). More specifically we aim to quantify population change, mortality and recruitment and to assess the status of recruited individuals. We also aim to relate recruitment and mortality to selected environmental factors reflecting water conditions, and to assess the relative importance of water conditions vs. human interference for the mortality of trees.

### Study area

**Figure 1:**
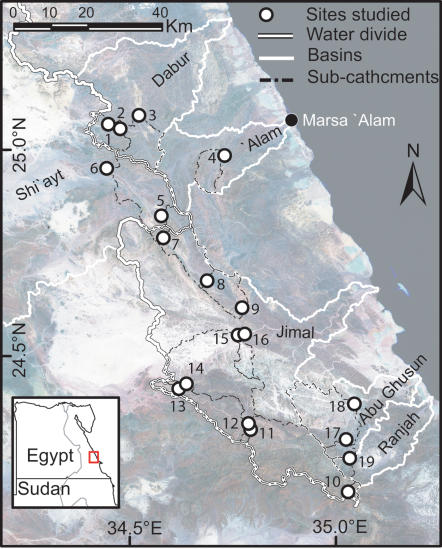
Study area. Studied sites in relation to basins, sub-catchments and east-west water divide. Each site is labelled with its ID (see Table 1). A Landsat TM image is displayed in the background.

The study area is located in the mountainous ED (Fig. 1). The ED ranks among the most extreme deserts in the world (the coefficient of variation of rainfall reaches 200%, mean annual precipitation <30 mm [5], [22]). Because gauge measurements are lacking and meteorological stations are few and scattered, studies on spatial and temporal distribution of water resources are rare (but see [25], [26]). Scattered showers, oreographic rain, dewfall, mist and fog are important water sources. Short and intense rainstorms generate floods in widespread dry river valleys, i.e. *wadis*, where soils have good infiltration capacity [25], [26]. Wadis intersect large areas of rock outcrops where run-off is high. Water reaching a site is therefore often related to its upper catchment size.

**Table 1:** Some characteristics of sites studied.

Sites	ID	Catch (km^2^)	Alt (m)	Coast (km)	Slope (%)	Area (ha)	Dens (ha^−1^)	Grn	Char
Dabur I	1	12	522	39	1.5	26	1.7	3	1
Dabur II	2	73	494	38	0.9	16	2.4	2	1
Dabur III	3	171	406	16	1.5	15	2.2	2	2
Sukkari	4	58	304	38	1.3	31	1.4	3	2
Hanjaliyyah	5	38	718	34	1.4	43	1.4	2	5
Kharrasha	6	213	570	32	0.9	27	1	2	0
Nuqrus U	7	11	598	38	4.3	5	8.4	3	2
Nuqrus M	8	116	415	41	1.6	182	0.4	2	6
Nuqrus	9	200	297	26	0.4	28	1.8	2	3
Hulus U	10	10	1130	34	2.2	3	9.3	1	11
Hulus M	11	5	673	39	2.1	4	7.8	2	0
Hulus M II	12	325	663	39	1.4	12	3.6	2	7
Gaetri	13	22	466	53	1.8	55	1.5	3	9
Hulus L	14	460	440	38	1.2	11	2.7	3	1
Jimal I	15	761	282	51	0.9	21	6.1	2	2
Jimal II	16	797	278	34	0.4	9	6.9	2	4
Sartut I	17	27	342	53	2.0	4	10.8	2	1
Abu Ghusun	18	222	216	36	2.0	15	3.5	3	7
Hulayfi	19	19	513	24	4.2	3	14.3	2	9

**ID** refers to labelling in Figs. 1 and 6. **Catch** is upper catchment size, **Alt** is altitude and **Dens** is mature tree density. Note that variation in **Dens** is considerable. **Grn** is the greenness category for the majority of individuals and **Char** is number of charcoal related observations.

Individuals of *Acacia tortilis* and *Balanites aegyptiaca* predominate in the wadis. They have high drought persistence because they send their roots deep into permanent soil moisture [27], [28]. Germination of seedlings and eventual recruitment of trees require surface moisture [11], but the main challenge for saplings is to survive until their roots have reached the deeper soil moisture.

Two subspecies of *A. tortilis* (Forssk.) Hayne occur in the ED according to Boulos [29]; i.e. ssp. *tortilis* and ssp. *raddiana* (Savi) Brenan. The majority of *A. tortilis* individuals recorded here are ssp. *raddiana* (also considered as a separate species: *A. raddiana* Savi); but since gradual morphological transitions are found and desert dwellers shape the very morphology of trees and bushes by their management strategies [6], we refer to *A. tortilis* at the species level.

The nomads inhabiting the study area are mainly Ababda, who with their animals live in and rely upon the wadis and their resources, particularly trees. Their pastoral nomadic lifestyle and management strategies are essential for environmental conservation; and the persistent influence of such strategies has shaped this hyper-arid landscape throughout millennia [6].

Charcoal production increases mortality when whole and alive (“green”) trees are used. According to traditional management customs in the ED, green trees should under no circumstances be felled [30]. People hesitate to admit breaking rules; but felling of green trees does occur and may be an act of “drought-induced despair”, be done by people without an attachment to the land or be a sign of cultural transformation [6], [16], [30], [31]. *Acacia tortilis* is the species mainly used for charcoal production in the ED due to its high quality wood [31]. Murray [17] described the production in the ED as follows, “Acacias are the trees usually sacrificed. The tree is dug right out by the roots, chopped up, and burnt for two or three days under a heap of earth.” This also describes the use of a kiln (*kamina*) for production, which is a method that works best with large logs and thick branches and even copes effectively with damp, “green” wood. *Kamina* production is a method probably introduced by the Romans in Antiquity [6]. Already in those times high energy requirements for blacksmiths, baths, mines and quarries in the area suggest high local production of charcoal [6], [30], [31]. Some authors report that ancient deforestation is still reflected in the landscape [28], [31]. This implies a very low recruitment, but recruitment has not yet been systematically studied (but see [31]).

## Materials and Methods

### Selection of sites and field data

In February and March 2003 we studied 19 sites located within and among several drainage systems in order to catch the topographical and hydrological variability in site conditions (Fig. 1 and Table 1). At each site a minimum of 30 individual trees was mapped and measured (for details see [32]). The height of individuals was estimated by a measuring stick (height<2 m) or by a clinometer. Vitality was subjectively estimated by “greenness” (0: no green leaves – 4: very dense canopy of vigorous leaves). Signs of sprouting, which has been recognized as a strategy to increase persistence in trees that grow in disturbed environments [11], [33]–[35], was recorded for each tree along with traces of pollarding and browsing. The presence of stumps, charcoal mines or other indicators of charcoal production were registered at each site.

Topographical and hydrological information for sites is derived in ESRI ® ArcGIS from the Shuttle Radar Topography Mission Digital Elevation Model (90 m resolution; http://www.jpl.nasa.gov/SRTM). Preprocessing, e.g. filling of holes, was done in Blackart 3.99 (http://www.terrainmap.com).

### Image analysis and change categorization

Imagery from the U.S. photo-reconnaissance satellite corona offers high resolution panchromatic panorama data between 1960 and 1972 [36]. corona is the only useable historical dataset available from the ED. Here we exploit the oldest (Dec. 1965) and best resolution (2.7 m at nadir) imagery available from the ED (KH-4a, mission 1027-1, frames 142–148). A high proportion of mature trees are recognizable in this imagery, and the potential for detailed change analyses is good [32]; see here for further information about imagery, preprocessing and interpretation of vegetation content.

**Figure 2:**
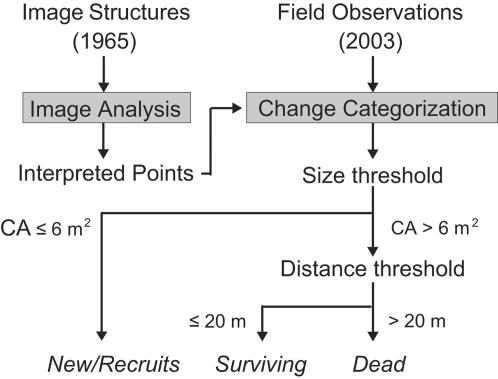
Flowchart. Image structures resembling trees are interpreted and represented as points. Together with trees recorded in field, all points are categorized as belonging to one of three Change Categories. In this process a size (Canopy Area of 6 m^2^) and distance (20 m) threshold was applied to compensate for GPS inaccuracies and limited image resolution [32]. Trees recorded in 2003 with CA ≤6 m^2^ are automatically categorized as *new*.

**Figure 3:**
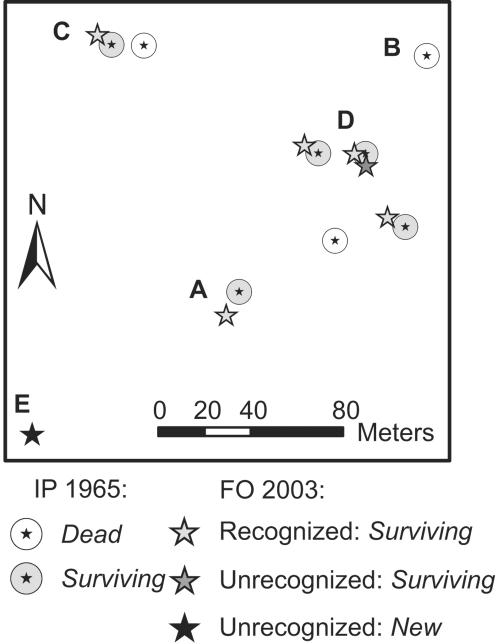
Change Categorization; an example from Wadi Gaetri. Distance is measured from each interpreted point (IP) to the nearest field observation (FO) with canopy area >6 m^2^. A If an IP has only one FO closer than 20 m, the two points are considered as referring to the same tree and categorized as *surviving*. B If an IP is separated by more than 20 m from any FO it is considered unmatched and categorized as *dead*. C If two IPs have the same FO closer than 20 m, the pair with the shortest distance is considered as referring to the same tree and the IP categorized as “*surviving*”. The second IP is categorized as *dead* if no other FO is closer than 20 m. Unmatched FOs are categorized as unrecognized and can be either D accounted for and merged with *surviving* or E not accounted for and merged with *new* (cf. [32]).

Structures interpreted as trees from the imagery (1965) were manually digitized as points. In a comparison of these interpreted trees (1965 imagery) with trees observed in 2003, three change categories result: *surviving* (recorded in 1965 and 2003), *dead* (recorded only in 1965) or *new* (recorded only in 2003; see Figs. 2 and 3). These categories are the basis for mortality, recruitment and change estimates.

### Recruitment, mortality, and change

The category *new* can reflect maximum recruitment after 1965. We estimate absolute recruitment, *N_ha_*, expressed as *new* per hectare, to account for the varying sizes of sites studied. We also estimate relative total recruitment, *N_t_*, expressed as the fraction of *new* in percentage of total (*dead*+*surviving*+*new*) observed population in the period studied (1965–2003). Annual rates will be referred to only for the purpose of comparison because there are no reasons to believe that recruitment (or mortality; see below) was temporally stable.

A *new* tree was not necessarily recruited during this period. Some larger *new* trees may well be older and have been present already in 1965, but not detectable on the image [32]. *Recruits* should be limited to young individuals, but age determination is problematic. Using size of individuals one must be aware that browsing suppresses growth, even for decades [11], [12], [14], [31]. Pollarding also affects growth and size of trees, though primarily mature trees [32]. Moreover, growth rate is unknown, but seems to be very slow [32].

To give a better recruitment estimate we adopt a 3 cm annual height increment to identify *recruits* in the period studied (37,33 years), i.e. all *new* individuals shorter than 112 cm, and thereby to calculate absolute (*R_ha_*) and relative total (*R_t_*) *recruitment* (defined as equivalent to *N_ha_* and *N_t_*, respectively). This is the best available estimate of height increment in a similar environment, i.e. Wadi Allaqi, ED, based on measures from ten unprotected *A. raddiana* seedlings of a 1988 cohort followed over 5 years by Springuel and Mekki [31]. They conclude that annual growth is less than 3 cm; but in fact growth shows great variability, and the mean is considerably lower (−22 cm; effect of browsing, to 26 cm, mean 0.8 cm).

For mortality, we calculate absolute (*M_ha_*) and relative total (*M_t_*) mortality (defined as equivalent to *N_ha_* and *N_t_*). We also calculate relative mature mortality, *M_m_*, expressed as the fraction of deaths in percentage of mature (*dead*+*surviving*) observed population in the period studied (1965–2003).

Absolute and relative change are investigated in a diagram where change is displayed as the difference between mortality and recruitment (based on *new* and *recruits*) and where sites with a similar type of change will cluster.

### Patterns in recruitment and survival

Since deserts are water-limited environments, water is a main factor influencing the presence and growth of trees. Several variables related to hydrological conditions (Table 2) were therefore selected to test for moisture-induced patterns in recruitment and survival. Possible human activities influencing mature tree survival, in particular charcoal production, are not tested in the model. A direct estimate of such influence is almost impossible to make because nomads do not talk freely about charcoal production, nor is it systematically monitored. Indirect measures of human influence such as access to an area are also difficult to quantify because movement patterns and family relations of the nomads should be taken into account. In addition one should also consider that their perception of the landscape differs from ours [30].

**Table 2:** Variables used as indicators of water conditions in models of recruitment and survival.

Variable	Type	Transformation	Interpretation
Catchment (km^2^)	R	Logarithmic base2	Calculated from SRTM DEM. Related to the amount of water reaching a site, both as surface water during flooding and as seeping subsurface soil moisture.
Mature density 1965 (ind/ha)	R	Logarithmic base2	Proxy for good growth conditions; i.e. in water-limited ecosystems it is reasonable to assume that this is related to good soil moisture conditions. However, it is also influenced by human intervention.
Coast (km)	R		Distance from coastline; indication of the probability of humid air reaching a site, e.g. occurrence of dew and oreographic rainfall. Humidity decreases at increasing distance.
Altitude (m asl)	R	Logarithmic base2	Correlated with temperature and therefore evapotranspiration, and with formation of dew and oreographic rain when interacting with coast; also correlated with catchment size for sites within the same basin.
Slope (%)	R/L	Logarithmic base2+1	At regional scale calculated as the slope of the wadi at the site in flow direction; at local scale calculated for each tree from the SRTM DEM. Indication of speed and infiltration of water run-off.
Thiessen polygons (m^2^)	L	Logarithmic base2+1	One polygon for each tree; each polygon has the unique property that any location within a region is closer to the region's tree than to the tree of any other region [44]. Inverse measure of density (cf. above).
Stream (m)	L	Square root	Distance from ephemeral stream as derived from the SRTM DEM; related to presence of surface moisture from flooding. Might have negative effect on survival on both short (uprooting) and long (no surface moisture; interacts with slope) distances.
Aspect (degrees)	L	Sinus	Calculated from SRTM DEM; related to solar radiation and evaporation pressure.

The variables are used either in the Regional (R; each site has one estimate) or Local (L; each tree has one estimate) models. Transformations are applied to achieve a normal distribution of variables. Aspect is sinus transformed to facilitate its interpretation. Outlying observations were removed from the dataset.

At a regional scale generalized linear models were fitted to a dataset in which each site represented one observation. For recruitment, modelled as counts per site of either *new* or *recruits*, we applied a model with a quasipoisson error structure (to allow for overdispersion) and a logarithmic link. Survival, i.e. the proportion survivors of the 1965 mature population (*surviving*+*dead*) per site, was modelled with a quasibinomial error structure (to allow for overdispersion) and a logit link. A backward elimination was performed starting with all covariates as main effects; subsequently the interactions were tested only for those main effects that were significant. This selection procedure was followed due to limited number of observations.

To test for local, within-site patterns in survival of trees caused by selected environmental factors (Table 2) the dataset of all *surviving* and *dead* trees was analyzed in a generalized linear mixed-effects model [37]. The site-factor was included as a random effect, while environmental factors were modelled as fixed effects.

## Results

### Recruitment, mortality and change

**Figure 4:**
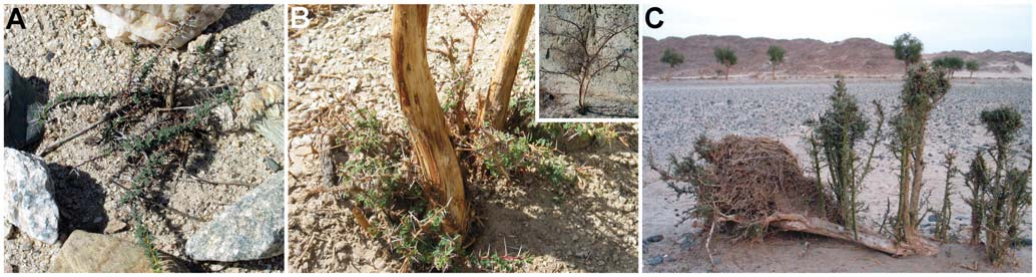
Sprouting. A Saplings frequently resprout after droughts and/or browsing. This individual has been intentionally encircled by stones. B An *Acacia tortilis* has to start all over again. C Also in *Balanites aegyptiaca* root and trunk sprouting seems to be an important mechanism.

In the field no seedlings and only a few small saplings were observed. Recruitment estimates for sites during the period considered range from extremely low values (1% at Gaetri) up to 38% (Table 3). At Abu Ghusun where recruitment was only 3% (*R_t_*) the smallest sapling found had been intentionally encircled by stones, probably for protection (Fig. 4A). The majority of *new* individuals is *A. tortilis*. The majority of *new* *B. aegyptiaca* individuals grows at Jimal I or II. All *new* (and *surviving*) individuals show signs of browsing. Of *new* individuals, 24% (3% of *recruits*) showed scars of former branches, and we frequently observed small individuals with dry branches. Most *new* individuals have greenness 2 (61%) or 1 (21%). In category 4 there is only 1 *new* (no *recruits*), while in category 0 there are 9 *new* individuals (8 of these are *recruits*; 3.2%). Of *new* individuals 95% (and 97% of *recruits*) showed signs of sprouting (Fig. 4). We also observed root sprouting in both *A. tortilis* and *B. aegyptiaca*. At Hanjaliyya where an *A. tortilis* had been dug out (cf. Study area) a small individual appeared from a surviving root. At the Jimal sites in particular *B. aegyptiaca* individuals tended to grow in rows, as if appearing from a root or fallen trunk beneath. Two uprooted, half burried indivduals observed there exemplify this, for their trunks and roots were covered with sprouts (Fig. 4C).

**Table 3:** Summary of change categorization for sites, including mortality and recruitment estimates.

Sites	Dead	Surviving			New			Recruits	Mortality			Recruitment			
		*Ac*	*Bal*	Σ	*Ac*	*Bal*	Σ		M_t_	M_m_	M_ha_	N_t_	N_ha_	R_t_	R_ha_
Dabur I	4	40		40	23	1	24	9	6	9	0.15	35	0.92	13	0.35
Dabur II	8	30		30	25		25	17	13	21	0.51	40	1.58	27	1.08
Dabur III	7	26		26	12		12	7	16	21	0.47	27	0.81	16	0.47
Sukkari	15	29		29	7		7	2	29	34	0.49	14	0.23	4	0.07
Hanjaliyyah	30	30		30	7		7	5	45	50	0.69	10	0.16	7	0.12
Kharrasha	12	14	2	16	27	1	28	15	21	43	0.44	50	1.04	27	0.56
Nuqrus U	16	26		26	14		14	6	29	38	3.01	25	2.63	11	1.13
Nuqrus M	42	24	1	25	9		9	6	55	63	0.23	12	0.05	8	0.03
Nuqrus	14	32	4	36	3	3	6	2	25	28	0.51	11	0.22	4	0.07
Hulus U	6	22		22	46		46	28	8	21	2.34	62	17.95	38	10.93
Hulus M	5	26		26	8		8	4	13	16	1.18	21	1.89	10	0.94
Hulus M II	16	27		27	5		5	2	33	37	1.29	10	0.40	4	0.16
Gaetri	44	36		36	6		6	1	51	55	0.80	7	0.11	1	0.02
Hulus L	8	22		22	10		10	8	20	27	0.72	25	0.90	20	0.72
Jimal I	87	7	34	41	1	32	33	18	54	68	4.10	20	1.56	11	0.85
Jimal II	32	4	26	30	1	21	22	5	38	52	3.69	26	2.54	6	0.58
Sartut I	8	35		35	3		3	1	17	19	1.85	7	0.69	2	0.23
Abu Ghusun	16	37		37	7		7	2	27	30	1.09	12	0.48	3	0.14
Hulayfi	14	29		29	3		3	3	30	33	4.43	7	0.95	7	0.95
Σ	384	496	67	563	217	58	275	141							
Total									31	41		23		12	

M_t_, M_m_, R_t_, and N_t_ are percentages, M_ha_, N_ha_ and R_ha_ are per hectare. *Ac* is *A. tortilis*, *Bal* is *B. aegyptiaca*. We recorded 838 individuals in the field. Of *surviving*, 382 were interpreted directly from imagery. In all 766 interpretations were done from imagery (*dead*+382).

All *surviving* individuals were pollarded, 29% showed sprouting and the vast majority fell into greenness category 2 (48%) and 3 (46%). Only 2 individuals were in category 4. For category 0, which might indicate natural mature mortality, 3 individuals (0.5%) were registered. One of these leafless individuals grew at Sartut I, and had evidence of burning at its foot. The other two grew in Hulus U where 73% of the mature individuals had a greenness of 1 (Table 1).

**Figure 5:**
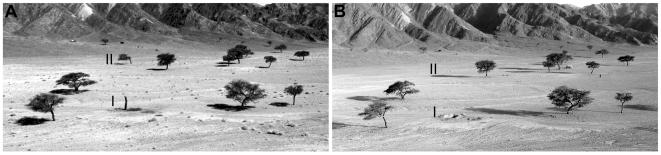
Changes in Wadi Gaetri between A 1996 and B 2003. I: In 2003 a hole in the ground is all that testifies to the presence of the stump (1996). II: A green tree has already lost half of its canopy. In 2003 this tree is completely gone, suggesting live felling for charcoal production.

We registered indications of charcoal production at all sites studied, except Hulus M and Kharrasha (Table 1). At some sites we also observed holes in the ground (Fig. 5), indicating that even roots had been dug out for charcoal production (cf. Study area). Mortality estimates ranged from very high (68%; M_m_ at Jimal I) down to 6% (M_t_ at Dabur I).

**Figure 6:**
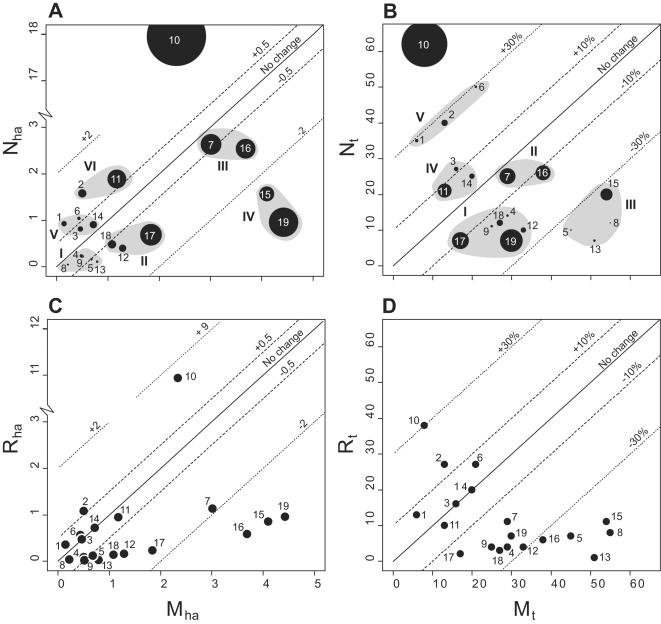
Trend of change for sites. Change, i.e. difference between mortality and recruitment, is the orthogonal distance from the diagonal axis. Labels are explained in Table 1. A and C show absolute change and B and D relative change. In A and B sizes of symbols are proportional to total vegetation density and recruitment estimates are based on *new* indivduals. Grey shapes show clusters of sites with similar types of change, resulting from similar combinations of mortality and recruitment: A I–III: low to intermediate negative, IV: high negative, V–VI: low to intermediate positive, B I–II: low to intermediate negative, III high negative, IV low positive and V high positive. In C and D recruitment estimates are based on *recruits*. Note the discontinuous scale on the y-axis in A and C.

The trend in population size for sites is seen in Fig. 6. For absolute change based on *N_ha_* and *M_ha_* six clusters of sites are apparent (Fig. 6A; numbers in parenthesis below refer to labelling). Hulus U (10) stands apart from these by its extremely positive change. For relative change based on *N_t_* and *M_t_* there are five clusters (Fig 6B). Again Hulus U shows a very high positive change, resulting from extremely high recruitment and low mortality.

When absolute change is based on *R_ha_* and *M_ha_*, only Hulus U and Dabur II (2) show a clear positive trend (Fig. 6C). The remaining sites in clusters V and VI (Fig. 6A) now exhibit nearly no change, except for Hulus M (11) which shows a negative trend. Sites in III and IV (Fig. 6A) become more similar, and all the rest of the negatively trending sites are now dominated by close to zero *R_ha_* and low to intermediate *M_ha_*.

For relative change based on *R_t_* and *M_t_* only 4 sites show a clearly positive trend, although much reduced (Fig. 6D). Dabur III (3) and Hulus L (14) show no change, Hulus M is now slightly negative, and the rest of the negatively trending sites cluster into two groups, corresponding to I and III (Fig. 6B).

### Patterns in recruitment and survival

**Figure 7:**
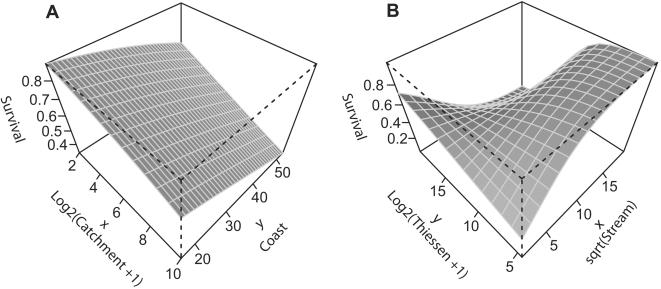
Predictions of the A regional and B local models of survival. For the interpretation of axes, note the transformation of variables. Note the viewing direction of B where the azimuthal direction is 315 degrees causing the swap of x and y axes.

None of the water related variables (Table 2) explain regional patterns in recruitment, based either on counts of *new* or *recruits*. For survival on a regional scale, the minimal adequate model consists of two significant main effects, catchment size, which is the strongest effect, and distance from the coast (Table 4 [Table pone-0000208-t005]). Both variables have a negative effect on survival of trees, e.g. for a given distance from the coast, the probability of survival is greatest for smaller catchments (Fig. 7A). The probability of survival is greatest on sites located at shorter distances from the coast and having small catchments. The adjusted R-squared for the model is 38% (Table 4).

**Table 4:** Summary statistics of the regional generalized linear model of survival.

	Estimate	SE	t-value	Pr(>|t|)	
(Intercept)	2.96	0.76	3.90	0.00	
Catchment	−0.17	0.06	−2.56	0.02	
Coast	−0.04	0.02	−2.42	0.03	
	Resid. DF	Resid. Dev	F	Pr(>F)	Adj. R^2^
NULL	18	132.15			
Catchment	17.00	99.31	7.51	0.01	0.2
Coast	16.00	72.73	6.08	0.03	0.38

Variables are transformed (Table 2). Dispersion parameter for quasibinomial family was estimated as 4.37. For Deviance (Dev.) terms are added sequentially (first to last). SE is Standard Error, SD is Standard Deviation, df is degrees of freedom and Adj. R^2^ is the adjusted coefficient of determination.

On a local scale, the minimal adequate model for survival consists of one significant main effect; distance from ephemeral stream (positive), and the significant interaction between distance from ephemeral stream and Thiessen polygons (negative; Table 5). For trees growing in small Thiessen polygons (less than 0.1 ha; corresponding to more than 9 trees/ha) survival increases at greater distances from an ephemeral stream (Fig. 7B). For trees growing in larger Thiessen polygons (lower tree densities), the effect of increasing distance from an ephemeral stream on survival is negative. There are two domains of low survival (<0.5); when both Thiessen polygons are small and distances from an ephemeral stream are short, and when both Thiessen polygons are large and distances are long.

**Table 5 pone-0000208-t005:** Summary statistics of the generalized linear mixed effects model of survival.

	Value	SE	DF	F-value	p-value	SD
(Intercept)	−2.22	1.55	960	2.05	0.153	0.89
stream	0.62	0.15	960	16.54	<0.001	
Thiessen	0.19	0.13	960	1.93	0.165	
stream:Thiessen	−0.05	0.01	960	12.05	0.001	
Residual						1.05

Variables are transformed (Table 2). Number of observations is 982, and number of groups 19. SE is Standard Error, SD is Standard Deviation and DF is degrees of freedom.

## Discussion

Estimates of overall recruitment in the ED are low (overall annual recruitment is between 0.31 (*R_t_*) and 0.60% (*N_t_*)), and it should be noted that these estimates are approximations and that even lower *recruitment* seems possible (cf. Materials and Methods). Probably it is the rarity of seedlings and small saplings that causes a desire among local people to preserve them (Fig. 4A). This rarity indicates a lack of recent large recruitment events in the ED. Even if an isolated rainfall event leads to the germination of seeds, seedling mortality can be close to 100% if the following weeks and/or seasons are dry, which is normally the case in hyper-arid environments [8], [13].

Although all *new* individuals were browsed and this suppresses their growth, it does not per se seem to increase mortality [13]. This is probably related to the great capacity of the trees studied to resprout. The process of recruitment seems to be one of repeated resprouting until one, or several, sprouts escape growth suppression (Fig 4). The frequency of sprouting in saplings makes it reasonable to question its long-term effect on the growth form of *A. tortilis* and its use to distinguish between sub-species [12], [29], [38].

Generally we did not investigate the type of sprouting observed [35]. The extent of root sprouting is therefore unknown, but we did observe clear examples in a few cases. This indicates that *recruits*, i.e. from seeds, may be even fewer than estimated here. If root-sprouting adds substantially to the long-term persistence of populations, the removal or destruction of a tree including its roots is of particular concern.

Although several studies report rare and scarce recruitment in arid lands, there exist few studies that estimate recruitment over longer time intervals and try to relate it to a hydrological regime. In Israel, however, Ben David-Novak and Schick [39] estimated recruitment (1972–1994) at two similar sites that varied only in surface water regime, and found higher values (0.57% vs. 2.35% annual recruitment) where surface flow was more frequent. In our models, however, none of the environmental factors tested could explain a significant part of the regional variability in recruitment, based on either *recruits* or *new* individuals. At our sites, the number of *recruits* recorded was generally very low, except at Hulus U. High recruitment there might result from its location close to large mountain massifs at high altitude (altitude had the strongest effect on both *new*; Pr = 0.19 and *recruits*; Pr = 0.06). Frequent small rainfall events, mist and dewfall are more probable at such sites and would improve soil moisture conditions and therefore promote high recruitment and tree density (Table 2 and see discussion below); and nomads do refer to special upstream conditions [30]. There is also a tendency for topographically similar sites, e.g. Nuqrus U and Hulayfi, to have high tree density and absolute recruitment (Tables 1 and 3).

Apparently, other variables should also be considered to explain regional patterns in recruitment. The effect of browsing on seedling and sapling mortality is one factor, but its continuous influence is difficult to assess and quantify in a field snapshot. Moreover, we have not considered the effects on seed germination caused by bruchid beetle infestation of seeds (negative) or by seed consumption by ungulates (positive) [13].

The lack of explanatory power of the models tested here can also indicate the importance of stochastic processes for recruitment. Water input in deserts is highly variable in space and time, and successive events which are needed for successful recruitment are extremely rare in hyper-arid environments. A simulation of recruitment frequency showed that only one large event every 50 years together with smaller intermediate events is needed to sustain desert tree populations of *A. raddiana* in the Negev [10]. That simulation was based on a higher mortality than what was found in the ED. Populations with lower mortality can be sustained with lower, i.e. less frequent, recruitment. A period of only 37 years is therefore probably too short to give a representative recruitment estimate for any of the sites studied.

Mature tree mortality in the ED has been substantial since 1965, at several sites 50% or higher. This corresponds to a total annual *M_m_* of 1.09% (*M_t_* of 0.84%), which is higher than the “natural mortality” recorded (0.5%). However, in our case being leafless is not a good indication of natural mortality. At Hulus U where two of these three individuals grew, we recorded a low night temperature (2°C) which might cause acacias to shed leaves [40]. A high proportion of trees had low greenness here (Table 1). When high mortality was first reported in the Negev, Israel, it was probably overestimated by misinterpreting trees having shed their leaves as dead trees [9]. There is also the problem of how long dead trees remain standing, which has to be known in order to calculate an annual rate. In the Negev it has been assumed that dead trees remain standing for 10 years [41]. Clearly, this indicates low/no charcoal production there today (although aggressive charcoal production was of concern there during the early 1930s [9]). Charcoal production is still ongoing in the ED. The burning at the foot of the last leafless individual suggest not only that this tree will not remain standing for long, but also that a charcoal producer induced its death [6].

Although standing, dead trees were hardly observed, remains of trees were, often together with traces of charcoal production. Note that since traces vanish with time absence at certain sites only indicates lack of recent production (Table 1). Several of these traces indicate *kamina* production (cf. Study area); and close to Sartut I, the burning itself was observed. The producer there was generally uncommunicative, particularly when we pointed out that his next tree was still alive. This tree still had a few leaves and was recently burnt at its foot.

Another sign of intensive production is the holes observed in the ground (Fig. 5) resulting from the exploitation of all parts of a tree, including accessible parts of the roots (cf. Study area). Acacia wood is extremely hard and difficult to cut, and digging out roots is also hard work. A full exploitation only makes sense from a commercial production point of view. The traditional, sustainable production for domestic use exploits only smaller, dry pieces of branches. In the Red Sea Hills, Sudan, the nomads have a separate method for such sporadic and minor production; the traditional *ferkabas* [6]. However, we have not observed this in the ED.

Signs of intensive production suggest that “green” trees are sacrificed (Fig. 5). We have never witnessed this, but nomads in the ED sometimes do admit cutting down viable trees when they decide they are “dead”, e.g. have exposed roots or no leaves. Moreover, one informant explained that a constrained economy forced people to overexploit trees in order to survive. For families in Wadi Allaqi charcoal production is their most important economic activity [42]; but nomads there insist they never cut down green trees or branches [31].

Based on the above discussion, human influence and charcoal production in particular seems to be an important variable influencing tree survival. If it could have been properly quantified (cf. Materials and Methods), we expect that it would explain a significant amount of the residual deviance in the regional model of survival. The final model was overdispersed, indicating that main explanatory variables were lacking [43]. Nevertheless, 38% of the variability in the dataset was explained by variables related to site water conditions (Tables 2 and 4).

On sites with large catchments located far from the coast (Fig. 7A) poor survival can be related to a lower probability of small rainfall events and dewfall. Water input from such events will mainly moisten the surface or shallow soil layers. At sites where this does not occur regularly, water for survival must be extracted from deeper soil layers. However, during long droughts when deep soil moisture is not replenished, trees will be more susceptible to water stress. It has been estimated that the growth of a small *Leptadenia pyrotechnica* shrub, with roots down to at least 11.5 m, can be sustained through (at least) four dry years [27]. Khusmaan Bedouins recognize that deeper-rooted acacias can survive a drought of at least twelve to fifteen years' duration [30]. Water stress over extended periods will eventually induce tree death. A strategy to withstand water stress is shedding of leaves, and this might furnish an excuse for tree felling for charcoal production even before the tree is really dead (cf. above).

At local scale, within sites, the model fitted also indicates that the probability of survival is related to the periodic presence of surface water, on a gradient across the wadi-profile. In this case a higher probability of death at short distances to an ephemeral stream indicates a destructive effect; i.e. uprooting caused by larger floods. Taking into account the significant interaction effect, however, shows that this pattern is only valid at high tree densities. Given low densities (large Thiessen polygons), survival is better at shorter distances. This switch can be explained if there is a correlation between Thiessen polygons and tree size, since one would expect that smaller trees with less developed root systems would be more susceptible to uprooting. However, this correlation has not been tested because at present the size of dead trees cannot be estimated accurately from the imagery [32]. High mortality at long distances from an ephemeral stream and low densities can be interpreted as indicating that the habitat is poor rather than being a site safe for uprooting. It should be noted that Thiessen polygons (insignificant effect) reflect not only natural growth conditions, but also past deforestation. Higher mortality at high and very low densities could also indicate a human desire to minimize the visual effect of their cutting. To test whether mortality is density dependent, point pattern methods can be applied [44], [45]. The fitted model should in all cases be interpreted with care for shorter distances because ephemeral streams have been derived from a 90 m resolution DEM.

### Conclusions

The overall trend in population size in that part of the ED studied here is negative. At some sites this negative trend is alarming because the reduction in mature trees is substantial (>50%) at the same time as recruitment is very low. However, there are also a few sites where the trend is positive and recruitment is better. Our frequent observations of sprouting in saplings indicate that this is an important mechanism to increase their persistence. It is the establishment itself that seems to be the main challenge in the recruitment process. There are indications that hydrological variables and surface water in particular can explain some of the observed patterns in mortality. However, our results indicate that direct human intervention, i.e. charcoal production, is the main cause of tree mortality.
